# Modification of surface glycan by expression of beta-1,4-N-acetyl-galactosaminyltransferase (B4GALNT2) confers resistance to multiple viruses infection in chicken fibroblast cell

**DOI:** 10.3389/fvets.2023.1160600

**Published:** 2023-07-06

**Authors:** Jin Se Park, Seung Je Woo, Chang Seon Song, Jae Yong Han

**Affiliations:** ^1^Department of Agricultural Biotechnology and Research Institute of Agriculture and Life Sciences, Seoul National University, Seoul, Republic of Korea; ^2^Avian Diseases Laboratory, College of Veterinary Medicine, Konkuk University, Seoul, Republic of Korea

**Keywords:** chicken, influenza A virus, B4GALNT2, genome editing, Newcastle disease virus

## Abstract

**Introduction:**

Infectious viruses in poultry, such as avian influenza virus (AIV) and Newcastle disease virus (NDV), are one of the most major threats to the poultry industry, resulting in enormous economic losses. AIVs and NDVs preferentially recognize α-2,3-linked sialic acid to bind to target cells. The human beta-1,4-N-acetyl-galactosaminyltransferase 2 (B4GALNT2) modifies α-2,3-linked sialic acid-containing glycan by transferring N-acetylgalactosamine to the sub-terminal galactose of the glycan, thus playing a pivotal role in preventing viruses from binding to cell surfaces. However, chickens lack a homolog of the *B4GALNT2* gene.

**Methods:**

Here, we precisely tagged the human *B4GALNT2* gene downstream of the chicken *GAPDH* so that the engineered cells constitutively express the human *B4GALNT2*. We performed a lectin binding assay to analyze the modification of α-2,3-linked sialic acid-containing glycan by human B4GALNT2. Additionally, we infected the cells with AIV and NDV and compared cell survivability, viral gene transcription, and viral titer using the WST-1 assay, RT-qPCR and TCID50 assay, respectively.

**Results:**

We validated human B4GALNT2 successfully modified α-2,3-linked sialic acid-containing glycan in chicken DF-1 cells. Following viral infection, we showed that human B4GALNT2 reduced infection of two AIV subtypes and NDV at 12-, 24-, and 36-hours post-infection. Moreover, cells expressing human *B4GALNT2* showed significantly higher cell survivability compared to wild-type DF-1 cells, and viral gene expression was significantly reduced in the cells expressing human *B4GALNT2*.

**Discussion:**

Collectively, these results suggest that artificially expressing human *B4GALNT2* in chicken is a promising strategy to acquire broad resistance against infectious viruses with a preference for α-2,3-linked sialic acids such as AIV and NDV.

## Introduction

Poultry, especially chicken, is a major source of protein, and there is a large demand for enhanced production of poultry products due to the growth of the global human population (1, 2). However, infectious diseases caused by viruses such as avian influenza virus (AIV) and Newcastle disease virus (NDV) threaten the poultry industry and food safety by infecting and spreading diseases in a huge number of poultry flocks, resulting in enormous economic losses (3). AIV frequently changes its antigenic properties via genetic mutations and reassortment, which makes it more difficult to develop effective preventative methods including vaccines (4). Therefore, it is important to develop novel strategies that can confer resistance against infection of broad subtypes of AIV and other pathogenic viruses such as NDV simultaneously.

An efficient strategy to suppress viral infection is to prevent virus binding to the target cell membrane by modifying the receptor molecule that is recognized by viruses. AIVs preferentially recognize sialic acids with the α-2,3 linkage to galactose, while human influenza viruses preferentially recognize sialic acids with the α-2,6 linkage (5–8). Moreover, the removal of α-2,3-linked sialic acid in target cells reduces the fusion of NDV to the cell membrane (9). Therefore, α-2,3-linked sialic acid is a common receptor molecule for various infectious viruses of poultry, and specific modification of α-2,3-linked sialic acid-containing glycans can confer multi-resistance against various infectious viruses including AIV and NDV.

B4GALNT2 is a glycosyltransferase that transfers N-acetylgalactosamine (GalNAc) to the sub-terminal galactose of α-2,3-linked sialic acid-containing glycans (10, 11). A recent study showed that expression of *B4GALNT2* prevents infection of influenza A viruses (IAVs) with a preference for α-2,3-linked sialic acid because the additional sugar GalNAc attached to the sub-terminal galactose hinders binding of hemagglutinin (HA) to the sialic acids by causing steric hindrance (12). NDV also recognizes α-2,3-linked sialic acid when it binds to target cells (9) and it is therefore assumed that *B4GALNT2* expression will confer resistance against NDV via a similar mechanism. However, most avian species, including chicken, lack a *B4GALNT2* gene in their genome, and the effects of *B4GALNT2* expression on AIV and NDV in chicken systems have not been validated.

Therefore, this study investigated whether B4GALNT2 confers viral resistance in a chicken system by artificially introducing the human *B4GALNT2* gene. By integrating the human *B4GALNT2* coding sequence at the 3′ end of the *GAPDH* gene linked via the T2A peptide coding sequence, we established *B4GALNT2*-expressing DF-1 chicken fibroblasts. Thereafter, we validated the modification of α-2,3-linked sialic acid-containing glycans and viral resistance of the engineered DF-1 cells. Our findings investigated the role of human B4GALNT2 against AIV and NDV in the chicken system for the first time and provided a potential strategy to render multi-disease-resistant chickens by introducing human *B4GALNT2* in the chicken genome.

## Materials and methods

### Construction of the GAPDH-B4GALNT2 tagging donor plasmid

For targeted tagging of the human *B4GALNT2* gene (NCBI Gene ID 124872) to the 3′ end of the *GAPDH* coding sequence (NCBI Gene ID 374193), the GAPDH-B4GALNT2 tagging donor plasmid containing intron 10 and exon 11 of *GAPDH*, T2A, the *B4GALNT2* coding sequence, and a bovine growth hormone polyadenylation site was synthesized (Bioneer, Daejeon, Korea) in the pBHA vector backbone. Thereafter, the human thymine kinase promoter and neomycin resistance gene were extracted from the *piggyBac* transposon expression vector (Addgene plasmid #92078, Watertown, MA, United States) and inserted into the GAPDH-B4GALNT2 tagging donor plasmid by digestion with AscI (New England Biolabs, Ipswich, MA, United States) and subsequent ligation.

### Construction of the CRISPR/Cas9 expression plasmid

The CRISPR/Cas9 vector targeting intron 10 of *GAPDH* was constructed using the PX459 vector (Addgene plasmid #62988). To insert guide RNA (gRNA) sequences into the CRISPR/Cas9 plasmid, sense, and antisense oligonucleotides were designed and synthesized (Bioneer). These oligonucleotides were annealed under the following thermocycling conditions: 30 s at 95°C, 2 min at 72°C, 2 min at 37°C, and 2 min at 25°C.

### Culture of chicken DF-1 fibroblasts

Chicken DF-1 fibroblasts [CRL-12203; American Type Culture Collection (ATCC), Manassas, VA, United States] were maintained and sub-passaged in Dulbecco’s minimum essential medium (DMEM; Hyclone Laboratories, Logan, UT, United States) supplemented with 10% fetal bovine serum (Hyclone Laboratories) and 1× Antibiotic-Antimycotic solution (Thermo Fisher Scientific, Waltham, MA, United States). DF-1 cells were cultured in an incubator at 37°C with 5% CO_2_ and 60–70% relative humidity.

### Transfection and antibiotic selection of DF-1 cells

To tag the human *B4GALNT2* gene to the *GAPDH* gene, the GAPDH-B4GALNT2 tagging donor plasmid (3 μg) and CRISPR/Cas9 expression vector (3 μg) were mixed with Lipofectamine 2000 reagent in Opti-MEM (Thermo Fisher Scientific), and 5 × 10^5^ DF-1 cells were treated with this mixture for 6 h at 37°C. After transfection, transfection mixtures were replaced with DF-1 culture medium, and G418 (300 μg/mL) was added to the culture medium 1 day after transfection. Selection is required for up to 7 days.

### Detection of tagging of B4GALNT2 to GAPDH in DF-1 cells

To identify tagging of *B4GALNT2* to *GAPDH*, genomic DNA was extracted from GAPDH-B4GALNT2 tagging DF-1 cells after G418 selection and analyzed using knock-in PCR analysis. The PCR conditions were as follows: 95°C for 5 min followed by 35 cycles of 95°C for 30 s, 60°C for 30 s, and 72°C for 30 s. Genomic regions encompassing the CRISPR/Cas9 target sites and *B4GALNT2* coding region were amplified with Integ F and Integ R primer sets (Table 1). The sample quality was checked using RT F and RT R primer sets (Table 1). The resultant PCR products were cloned into the pGEM-T Easy Vector (Promega, Madison, WI, United States) and sequenced using an ABI Prism 3030XL DNA Analyzer (Thermo Fisher Scientific). The sequence was compared against assembled genomes using the Basic Local Alignment Search Tool (BLAST).

**Table 1:** Primers used in this study.

Primers	Sequence
Integ F	5’-GCAGTTCTGGTGCGGTTCTG-3′
Integ R	5’-GGAGTCCCCGAGACACATTC-3′
RT F	5’-GGGTGCTGGCATTGCACTGA-3′
RT R	5’-GGTGAAGAGCAGGGGCTCCA-3′
GAPDH #1 sg RNA F	5’-CACCGCTATTCCTTATAAAGAAAGT-3′
GAPDH #1 sgRNA R	5’-AAACACTTTCTTTATAAGGAATAGC-3’
B4GALNT2 qRT F1	5’-GGACACTGAACACCCTTGCT-3’
B4GALNT2 qRT R1	5’-CTCTCTGGTCCAGGGTCGTA-3’
IAV M gene qRT F	5’-AACCGAGGTCGAAACGTACG-3’
IAV M gene qRT R	5’-CGGTGAGCGTGAACACAAAT-3’
NDV M gene qRT F	5’-GGCACGAGCTACTCTCTTCC-3’
NDV M gene qRT R	5’-CTCCCAGGGATCTTTTCCGG-3’

### Analysis of B4GALNT2 expression in GAPDH-B4GALNT2 tagging DF-1 cells

Total RNA was isolated from wild-type and GAPDH-B4GALNT2 tagging DF-1 cells using TRIzol reagent (Thermo Fisher Scientific) and reverse-transcribed using the Superscript III First-strand Synthesis System (Thermo Fisher Scientific). cDNA from wild-type and GAPDH-B4GALNT2 tagging DF-1 cells was amplified with primers specific for exon 10 of *GAPDH* and the *B4GALNT2* coding sequence. The PCR conditions were as follows: 95°C for 5 min followed by 35 cycles of 95°C for 30 s, 60°C for 30 s, and 72°C for 30 s. The total RNA was also isolated from GAPDH-B4GALNT2 single DF-1 clone (clone #12) and reverse-transcribed as mentioned above. cDNA from clone #12 and wild-type DF-1 cells was subject to RT-qPCR using a StepOnePlus real-time PCR system (Thermo Fisher Scientific) using the primer set listed in Table 1. The detailed PCR method is described in a previous study (13). RT-qPCR was performed in triplicate, and relative quantification of the target gene was normalized to that of the chicken *beta-actin* gene.

### Lectin binding assay

The lectin-binding assay was conducted as previously reported (12). For flow cytometry, cultured GAPDH-B4GALNT2 tagging and wild-type DF-1 cells were trypsinized and washed with 1 × PBS. Thereafter, cells were treated with green fluorescent *Maackia Amurensis* (MAA) Lectin I (20 μg/mL; Vector Laboratories FL-1311, Burlingame, CA, United States) for 20 min at 4°C, washed in 1 × PBS, and analyzed. For microscopy, cells were trypsinized, washed with 1 × PBS, placed on HistoBond microscope slides (Paul Marienfeld, Lauda-Konigshofen, Germany), and dried overnight at room temperature. After hydration, cells were treated with MAA-lectin I (20 μg/mL) for 1 h at room temperature, washed three times with 1 × PBS, mounted using VECTASHIELD Antifade Mounting Medium with DAPI (Vector Laboratories H-1200), and imaged under a fluorescence microscope.

### IAVs

PR8-H5N8 viruses were generated using reverse genetic systems from eight bidirectional PHW2000 plasmids encoding the *PB1*, *PB2*, *PA*, *HA*, *NA*, *NP*, *NS*, and *M* genes and PR8-H9N2 low pathogenicity viruses kindly gifted by Prof. Song Chang Seon of Konkuk University, South Korea. Viruses were rescued by co-transfection of the eight bidirectional plasmids into co-cultured Madin-Darby canine kidney cells (MDCK; ATCC, CCL-34) and human 293 T embryonic kidney cells (ATCC, CRL-11268). The generated viruses were grown in MDCK infection medium, which consisted of DMEM (Hyclone Laboratories) supplemented with 0.3% bovine serum albumin (BSA; Sigma-Aldrich, MO, United States), 1 × antibiotic-antimycotic reagents (Thermo Fisher Scientific), and 1 μg/mL TPCK-treated trypsin (Sigma-Aldrich), and then incubated at 37°C for 48 h. The virus stocks were further propagated in 10-day-old embryonated chicken eggs. Aliquots of infectious viruses were stored at −80°C for further experiments. All work with low pathogenicity viruses was conducted in a biosafety level 2 facility approved by the Institutional Biosafety Committee, Seoul National University.

### NDV

NDV (B1 type, B1 strain) was kindly gifted by Prof. Kwon Hyuk-Joon of Seoul National University, South Korea. The virus was further propagated in 10-day-old embryonated chicken eggs. Aliquots of infectious viruses were stored at −80°C for further experiments. All works with low pathogenicity viruses were conducted in a biosafety level 2 facility approved by the Institutional Biosafety Committee, Seoul National University.

### Viral titration of infected cells

Viral titrations of IAV- and NDV-infected cells were performed using MDCK and DF-1 cells (ATCC, CRL-12203), respectively, to determine the median tissue culture infectious dose (TCID_50_). In brief, supernatants of infected cells were used to infect confluent layers of MDCK (for IAV titration) or DF1 (for NDV titration) cells in 96-well plates with serum-free DMEM supplemented with 0.3% BSA, 1% penicillin/streptomycin (Thermo Fisher Scientific), and 1 μg/mL TPCK-trypsin. Serial dilutions of the supernatant were added to five wells of a 96-well culture plate in triplicate. After 72–96 h, the cytopathic effects were observed and quantified via crystal violet (Sigma-Aldrich) staining. The TCID_50_ values per ml were calculated using the Spearman-Karber formula (14).

### Measurement of viral gene expression of infected cells

Total RNA was extracted from wild-type, human *B4GALNT2* expressing #12 clone 6 h post-infection of IAVs (H5N8, H9N2), NDV and reverse-transcribed to synthesize cDNA as mentioned above. The cDNA was subject to RT-qPCR as mentioned above using primer sets listed in Table 1. The gene expression was normalized by chicken beta-actin (*ACTB*, NCBI Gene ID 396526). The detailed procedure was described in a previous study (15).

### Statistical analysis

Statistical analysis was performed using GraphPad Prism software (GraphPad Software, United States). Significant differences between groups were determined by the paired *t*-test. *p* < 0.05 indicated statistical significance.

## Results

### Establishment of human B4GALNT2-expressing DF-1 cells

**Figure 1:**
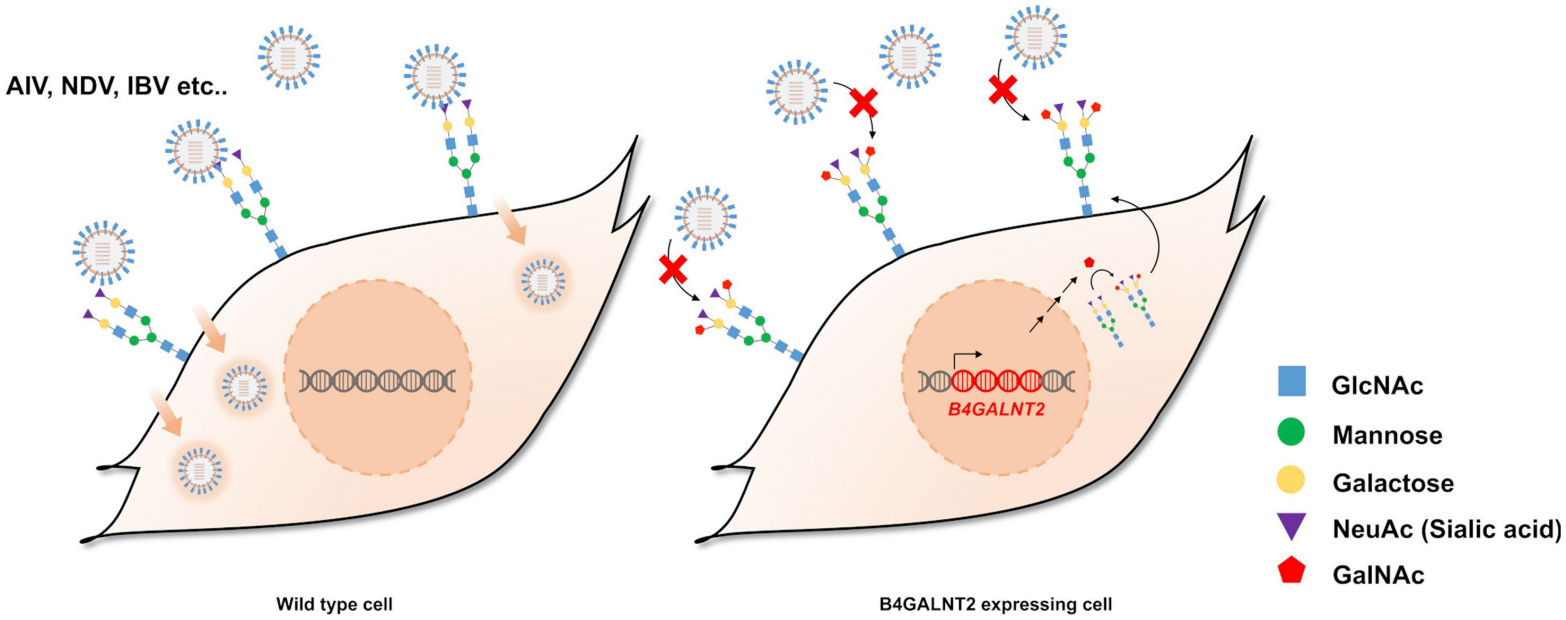
Schematic illustration of the study and expression of *B4GALNT2* in chicken cells. Illustration of resistance to virus infection conferred by B4GALNT2. In wild-type DF-1 cells, viruses recognize α-2,3-linked sialic acid, bind to the cell membrane, and subsequently infect target cells. In B4GALNT2-expressing DF-1 cells, α-2,3-linked sialic acid-containing glycans are modified by the addition of GalNAc and this prevents the binding of infectious viruses such as AI and NDV.

**Figure 2:**
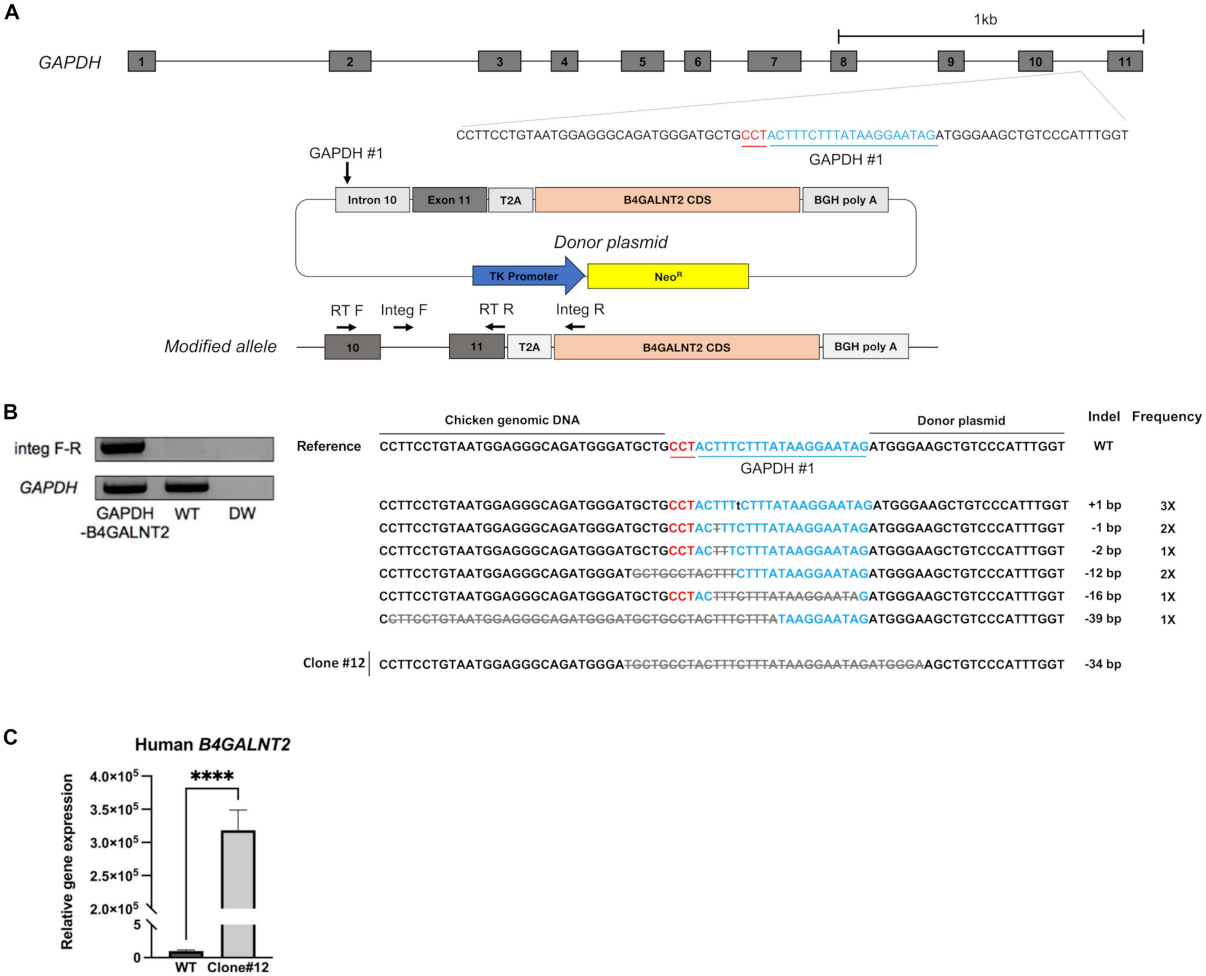
Establishment of GAPDH-B4GALNT2 tagging DF-1 cells. **(A)** Schematic illustration of tagging of *B4GALNT2* to *GAPDH*. **(B)** Integration of the donor plasmid into the target site was validated by knock-in PCR analysis using Integ F, R primer sets and sequencing analysis through the TA cloning method. The sample quality was checked by PCR analysis using RT F, R primer sets. The single B4GALNT2-GAPDH clone #12 was established, and the integration of donor plasmid was analyzed through sequencing. Red letters indicate the protospacer adjacent motifs (PAM) sequence and blue letters with GAPDH #1 refer to the guide RNA sequence. Gray letters with lines indicate deletions and lowercase black letters indicate insertions. Indel mutations are presented. **(C)** Comparison of human *B4GALNT2* gene expression level through RT-qPCR. The data are presented as the mean ± standard deviation (*n* = 3). Data were analyzed using a student’s *t*-test. ****p < 0.0001.

B4GALNT2 is an enzyme that adds GalNAc to the sub-terminal galactose of α-2,3-linked sialic acid-containing glycans and can therefore confer resistance against infection of viruses with a preference for α-2,3-linked sialic acid (Figure 1) (12). To express the *B4GALNT2* gene in chicken cells, the human *B4GALNT2* coding sequence was tagged to the 3′ end of the *GAPDH* gene using CRISPR/Cas9-mediated genome editing. The constructed donor vector contained intron 10 and exon 11 of the *GAPDH* gene, which contained the gRNA (GAPDH#1) targeting site, followed by T2A and the human *B4GALNT2* coding sequence (Figure 2A). When the donor vector and CRISPR/Cas9 plasmid targeting intron 10 of the *GAPDH* gene were co-transfected, the donor vector could be inserted into the target site by the non-homologous end joining (NHEJ)-mediated DNA repair pathway without altering *GAPDH* gene expression (Figure 2A). By transfecting the donor vector and CRISPR/Cas9 plasmid and performing subsequent drug selection, the GAPDH-B4GALNT2 tagging DF-1 cell line was successfully established. The integration in the forward direction at the target site was detected in GAPDH-B4GALNT2 tagging DF-1 cell line by PCR analysis using Integ F and R primer sets (Table 1). The sequencing analysis of the GAPDH-B4GALNT2 tagging DF-1 cell line was subsequently performed to confirm the integration of the donor vector at the target site (Figure 2B). To exclude DF-1 cells in which the donor vector was integrated into the reverse direction at the target site, single clones of the GAPDH-B4GALNT2 tagging DF-1 cell line were cultured and the DF-1 clone (Clone#12) was selected. The donor vector was validated to be integrated into the forward direction at the target site in clone #12 by sequencing analysis (Figure 2B). The human *B4GALNT2* mRNA expression was significantly higher than control wild-type (WT) DF-1 cells (Figure 2C).

### Modification of α-2,3-linked sialic acid-containing glycans by human B4GALNT2 expression

**Figure 3:**
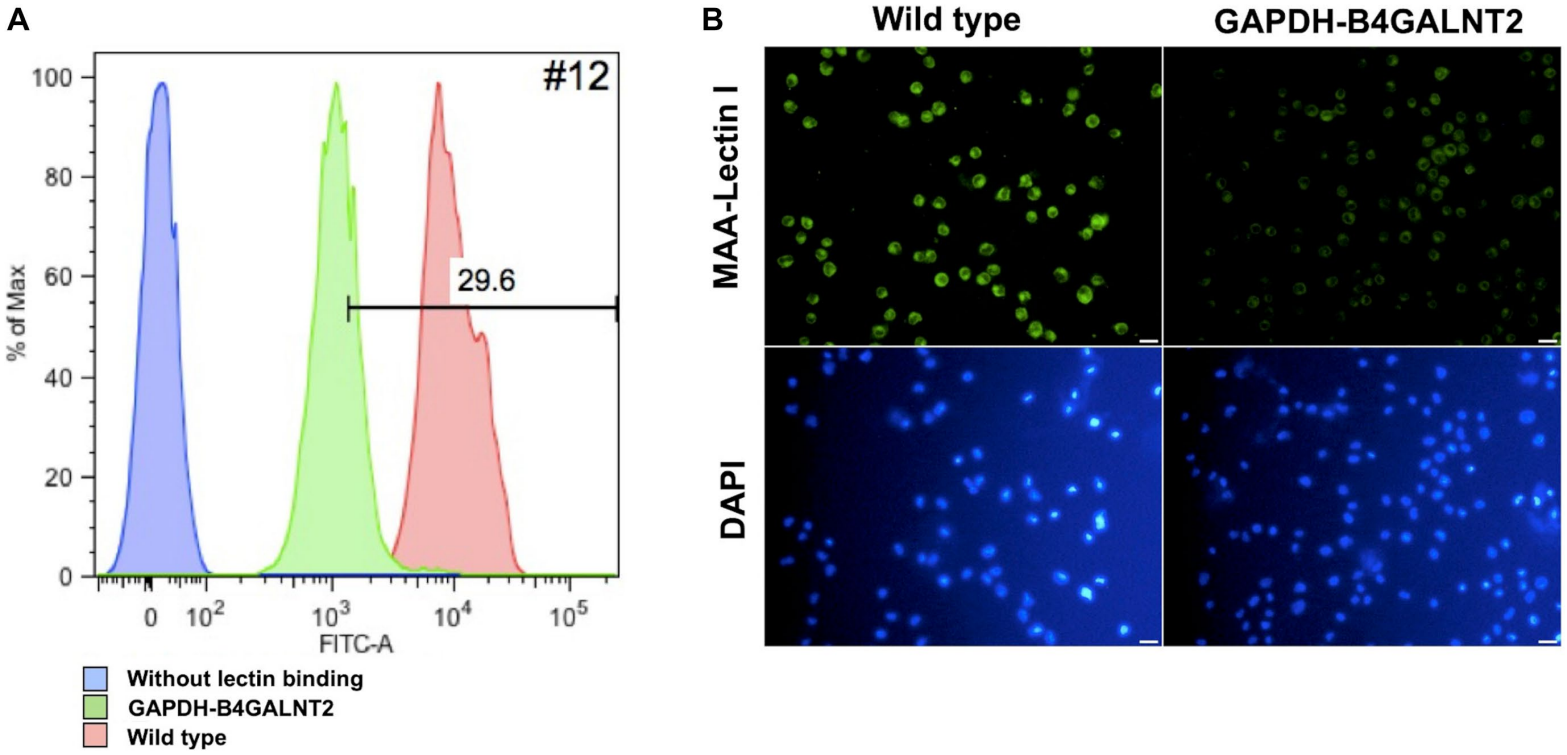
Lectin binding assay. **(A)** Flow cytometry histograms showing binding of green fluorescent MAA-lectin I to DF-1 clone #12. The lectin binding affinity was lower in GAPDH-B4GALNT2 tagging DF-1 clone #12 than in wild-type DF-1 cells. Red is wild-type DF-1 cells, green is GAPDH-B4GALNT2 tagging DF-1 cells, and blue is a negative control without lectin binding. **(B)** Green fluorescent MAA-lectin I was applied to DF-1 clone #12 and detected under a fluorescence microscope. Wild-type DF-1 cells were used as a control. Scale bar = 50 μm.

To determine whether human B4GALNT2 modifies α-2,3-linked sialic acid-containing glycans in chicken DF-1 cells, we treated GAPDH-B4GALNT2 tagging DF-1 clone #12 with green fluorescent labeled MAA-lectin I, which binds to α-2,3-linked sialic acid. Flow cytometry confirmed that the lectin binding affinity was significantly lower in clone #12 than that in wild-type DF-1 cells (Figure 3A). We also confirmed that the intensity of fluorescence of MMA-lectin I was lower in clone #12 compared with wild-type DF-1 cells under a fluorescence microscope (Figure 3B). These results showed that human B4GALNT2 is functional and modifies α-2,3-linked sialic acid-containing glycans in chicken cells.

### Expression of B4GALNT2 reduces viral infection of chicken DF-1 cells

**Figure 4:**
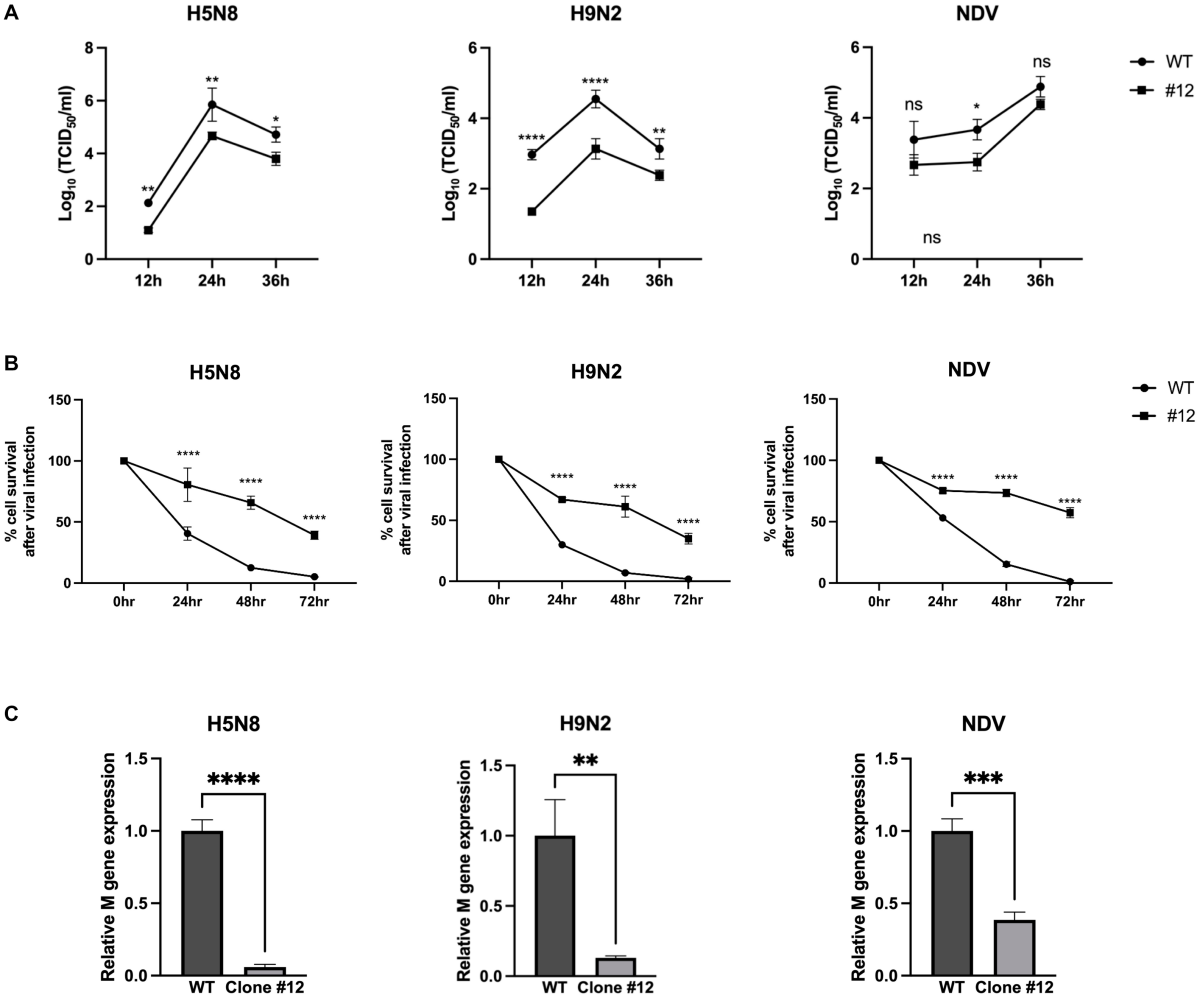
The challenge of GAPDH-B4GALNT2 tagging DF-1 cells with AIV and NDV. **(A)** Wild-type DF-1 cells and DF-1 clone #12 were infected with H5N8, H9N2, and NDV [Multiplicity of infection (MOI) of 0.1]. Viral titers were determined using TCID_50_ assays after 12, 24, and 36 h of infection. **(B)** Survival rates of wild-type DF-1 cells and DF-1 clone #12 were measured using WST-1 reagent after 24, 48, and 72 h of infection of H5N8, H9N2, and NDV. **(C)** Viral M gene expression of wild-type DF-1 cells and clone #12 was measured after 6 h post-infection through RT-qPCR. Data were analyzed by paired t-test. *p < 0.05, **p < 0.01, ***p < 0.005, ****p < 0.0001.

To investigate whether expression of human *B4GALNT2* efficiently reduces viral infection of DF-1 cells, DF-1 clone #12 was infected with two subtypes of low pathogenicity avian influenza (LPAI); H5N8 and H9N2 as well as NDV [Multiplicity of infection (MOI) of 0.1]. After 12-, 24-, and 36-h of infection, the viral titer was measured by the TCID_50_ assay. The viral titer of IAVs (H5N8, H9N2) was significantly lower in DF-1 clone #12 than in wild-type DF-1 cells at 12-, 24- and 36-h post-infection (hpi). The viral titer of NDV was significantly reduced in DF-1 #12 clone compared to wild-type DF1 cells at 24-hpi, while viral titer of NDV at 12- and 36-hpi showed non-significant reduction (*p* = 0.0503 at 12-hpi and *p* = 0.2159 at 36-hpi respectively) (Figure 4A). Additionally, the cell survival rate was determined at 24-, 48-, and 72-hpi. Following H5N8, H9N2, and NDV infection, the survival rate of DF-1 clone #12 was significantly higher than that of wild-type DF-1 cells (Figure 4B). To further analyze the virus resistance of clone #12, we measured viral gene (M gene) expression at 6-hpi. The results showed that viral M gene expression of IAVs (H5N8, H9N2) and NDV in clone #12 was significantly lower than that in wild-type DF-1 cells (Figure 4C). Collectively, these results showed that expression of the human *B4GALNT2* gene efficiently conferred resistance against various infectious viruses with a preference for α-2,3-linked sialic acid.

## Discussion

Infectious viruses are a major threat to the poultry industry. The wide and rapid spread of viral disease can cause huge economic losses owing to high mortality. For example, during an AIV outbreak in the United States in 2014–2015, the estimated economic losses were nearly $3.3 billion and approximately 50.4 million hens were euthanized (16, 17). Moreover, the isolation of new NDV strains and NDV outbreaks are continuously reported worldwide, and such outbreaks have caused significant economic losses (18–21). Therefore, prevention of viral diseases is a major goal in the poultry industry. AIVs preferentially recognize α-2,3-linked sialic acid to bind to the target cell membrane (5–8). In addition, NDV recognizes α-2,3-linked sialic acid when it infects target cells (9). Therefore, specific modification of α-2,3-linked sialic acid-containing glycans is expected to hamper viral binding and confer broad resistance against viruses recognizing α-2,3-linked sialic acids such as AIV and NDV. In a pioneering study, B4GALNT2 was identified as a pan-AIV restrictive host factor because it specifically modifies α-2,3-linked sialic acid-containing glycans by attaching GalNAc to the sub-terminal galactose (12). However, *B4GALNT2* is not present in the chicken genome; therefore, human *B4GALNT2* was artificially introduced into chicken DF-1 cells to hinder the binding of viruses to the target cells.

In this study, it was observed that the expression of human B4GALNT2 in DF1 cells significantly reduced lectin binding, although it did not completely block the binding, which is also reported in another study where an MDCK cell line overexpressing *B4GALNT2* was established (22). Heldt et al. demonstrated that inhibiting viral entry, including fusion, endocytosis, and binding to the cells, was less successful in reducing the peak viral titer. However, it delayed viral infection by up to 50 h, providing more time for immune cells to counteract the viral infection. Inhibiting viral RNA transcription or viral protein synthesis showed the most significant reduction in viral replication, although some cells still became infected (23). In our results, the expression of *B4GALNT2* in DF1 cells hindered virus binding to the cells and significantly reduced viral replication. However, complete inhibition was not achieved due to partial virus binding to the cells and the fact that B4GALNT2 interferes with viral entry step less successful in reducing the viral production. Despite this, the expression of human *B4GALNT2* led to higher cell survivability, possibly due to decreased virus binding and subsequent reduced viral replication. The reduced virus binding could also allow more time for immune cells to counteract against viruses and reduce excessive inflammation, which can be fatal to the infected animals (24). Furthermore, the modification of α-2,3-linked sialic acid by B4GALNT2 could inhibit the binding of infectious bronchitis virus (IBV) to the host, as IBV also binds to α-2,3-linked sialic acid during infection (25). Therefore, the expression of *B4GALNT2* has the advantage of simultaneously reducing the initial binding of multiple viruses to the host. Further studies are needed to analyze the resistance of human *B4GALNT2*-expressing DF-1 cells against IBV infection. Collectively, combining the expression of human *B4GALNT2* with inhibiting viral RNA or protein synthesis could render chickens resistant to multiple virus infections in two ways: B4GALNT2 reduces the initial binding of multiple viruses to cells, increasing cell survivability, and blocking viral RNA or protein synthesis efficiently inhibits viral replication.

In our results, the expression of human B4GALNT2 in DF1 cells significantly reduced the growth of NDV up to 24-hpi, although the inhibitory effect on NDV was reduced at 36-hpi. It has been reported that NDVs use both α-2,3-linked sialic acids and α-2,6-linked sialic acids during infection (26). Meanwhile, B4GALNT2 specifically hinders the binding of viruses to α-2,3-linked sialic acids (12, 22). Therefore, NDVs can still infect host cells in the presence of human B4GALNT2 by utilizing the α-2,6-linked sialic acids that are not interfered with by B4GALNT2. To completely inhibit NDV binding to cells, it is expected that modification of both α-2,3-linked sialic acids and α-2,6-linked sialic acids can be achieved, for example, by expressing both *B4GALNT2* and *Photobacterium* sp. α-2,6-sialyltransferase pseudosialidase, which specifically removes α-2,6-linked sialic acids (27).

To compare viral M gene expression between *B4GALNT2*-expressing DF1 cells and wild-type DF1 cells, the M gene expression was normalized using the chicken beta-actin (*ACTB*) gene instead of the chicken glyceraldehyde-3-phosphate dehydrogenase (*GAPDH*) gene (NCBI Gene ID 374193). It has been reported that the expression of the *GAPDH* gene was significantly affected when infected by influenza viruses in human T cells, whereas the expression of *ACTB* did not change (28). Additionally, the *GAPDH* gene has been shown to be affected by viral infections such as SARS-CoV-2 (29). Therefore, the chicken *ACTB* gene was used an internal control for measuring viral M gene expression instead of the chicken *GAPDH* gene.

To develop and maintain chicken lines with viral resistance, it is critical to maintaining resistance at a consistent level after these lines are generated. Therefore, when viral resistance is conferred by introducing a restrictive host factor, this factor must be stably and consistently expressed. Moreover, uniform expression of restrictive host factors in organs, especially the lungs, is essential to inhibit viral infection. However, the CMV and RSV promoters, which are commonly used for transgene expression in eukaryotes, cannot guarantee the consistent and uniform expression of transgenes because CpG methylation of these promoters occurs in varying degrees in each organ of transgenic chickens (30–32). Silencing of the CMV promoter by hyper-methylation has been reported in other animals (33–35). Furthermore, random integration and copy number variation of *piggyBac* transposons hamper the exact quantification of the expression levels of integrated exogenous factors (36). In this regard, targeted tagging of restrictive host factors to the *GAPDH* gene by CRISPR/Cas9-NHEJ can be used to consistently and uniformly express restrictive host factors.

Recently, Madin-Darby canine kidney (MDCK) cells expressing human *B4GALNT2* were reported and expressing human *B4GALNT2* hindered influenza virus entry except for A/WSN/33 (WSN) strain which showed the binding capability to α-2,6-linked sialic acid. This model was designed to screen for influenza viruses that require α-2,3-linked sialic acid (22). Although the integration of human *B4GALNT2* into the cells is similar to this study, our purpose is to establish a multi-poultry disease-resistant chicken cell line. We also found that human *B4GALNT2* in chicken cells significantly reduced NDV infection. In addition, while dogs possess the endogenous *B4GALNT2* gene (NCBI gene ID: 491067), chickens lack the *B4GALNT2* gene. Therefore, the inhibitory effect of virus infection in engineered chicken cells could be dominantly mediated by human *B4GALNT2* expression. Furthermore, while the human *B4GALNT2* gene was expressed by a lentiviral vector in MDCK cells, the engineered chicken DF-1 cells in this study constitutively expressed human *B4GALNT2* by precisely tagging the gene to the *GAPDH* gene. Based on our study, multi-disease-resistant chickens expressing human *B4GALNT2* are expected to be produced in the near future.

In conclusion, we successfully developed chicken DF1 cells constitutively expressing human *B4GALNT2,* which was tagged into the *GAPDH* gene. The expression of human B4GALNT2 in these cells led to significant reductions in virus binding, viral gene expression, and viral titer, ultimately resulting in higher cell survivability. Although the expression of human *B4GALNT2* did not completely block viral infection, it demonstrated the ability to suppress multiple viruses, including AIV and NDV, with a preference for α-2,3-linked sialic acid. These findings highlight the potential of introducing human *B4GALNT2* into chicken using genome editing technology as a promising approach to reduce virus infections. Furthermore, targeting other host proteins involved in viral RNA or protein synthesis in combination with B4GALNT2 could contribute to development of multiple virus-resistant chickens in the near future.

## Data availability statement

The original contributions presented in the study are included in the article/supplementary material, further inquiries can be directed to the corresponding author.

## Author contributions

JP and SW participated in the design of the study, conducted the experiments, interpreted the data, and wrote the draft of the manuscript. CS participated in the conceptualization and revision of the manuscript. JH participated in the writing of the final version of the manuscript, a conception of the work, and overall coordination. All authors reviewed the manuscript and approved the final manuscript.

## Funding

This work was supported by the National Research Foundation of Korea (NRF) grant funded by the Korea government (MSIP) [NRF-2015R1A3A2033826].

## Conflict of interest

The authors declare that the research was conducted in the absence of any commercial or financial relationships that could be construed as a potential conflict of interest.

## Publisher’s note

All claims expressed in this article are solely those of the authors and do not necessarily represent those of their affiliated organizations, or those of the publisher, the editors and the reviewers. Any product that may be evaluated in this article, or claim that may be made by its manufacturer, is not guaranteed or endorsed by the publisher.
